# The Impact of C-3 Side Chain Modifications on Kynurenic Acid: A Behavioral Analysis of Its Analogs in the Motor Domain

**DOI:** 10.3390/ijms25063394

**Published:** 2024-03-16

**Authors:** Diána Martos, Bálint Lőrinczi, István Szatmári, László Vécsei, Masaru Tanaka

**Affiliations:** 1 HUN-REN-SZTE Neuroscience Research Group, Hungarian Research Network, University of Szeged, Danube Neuroscience Research Laboratory, Tisza Lajos krt. 113, H-6725 Szeged, Hungary; martos.diana@med.u-szeged.hu; 2 Institute of Pharmaceutical Chemistry and HUN-REN–SZTE Stereochemistry Research Group, University of Szeged, Eötvös u. 6, H-6720 Szeged, Hungary; lorinczi.balint@szte.hu (B.L.); szatmari.istvan@szte.hu (I.S.); 3 Department of Neurology, Albert Szent-Györgyi Medical School, University of Szeged, Semmelweis u. 6, H-6725 Szeged, Hungary

**Keywords:** tryptophan, kynurenic acid, analogs, blood–brain barrier, drug design, drug delivery, exploratory behavior, motor skills, emotions, neuropsychiatry

## Abstract

The central nervous system (CNS) is the final frontier in drug delivery because of the blood–brain barrier (BBB), which poses significant barriers to the access of most drugs to their targets. Kynurenic acid (KYNA), a tryptophan (Trp) metabolite, plays an important role in behavioral functions, and abnormal KYNA levels have been observed in neuropsychiatric conditions. The current challenge lies in delivering KYNA to the CNS owing to its polar side chain. Recently, C-3 side chain-modified KYNA analogs have been shown to cross the BBB; however, it is unclear whether they retain the biological functions of the parent molecule. This study examined the impact of KYNA analogs, specifically, SZR-72, SZR-104, and the newly developed SZRG-21, on behavior. The analogs were administered intracerebroventricularly (i.c.v.), and their effects on the motor domain were compared with those of KYNA. Specifically, open-field (OF) and rotarod (RR) tests were employed to assess motor activity and skills. SZR-104 increased horizontal exploratory activity in the OF test at a dose of 0.04 μmol/4 μL, while SZR-72 decreased vertical activity at doses of 0.04 and 0.1 μmol/4 μL. In the RR test, however, neither KYNA nor its analogs showed any significant differences in motor skills at either dose. Side chain modification affects affective motor performance and exploratory behavior, as the results show for the first time. In this study, we showed that KYNA analogs alter emotional components such as motor-associated curiosity and emotions. Consequently, drug design necessitates the development of precise strategies to traverse the BBB while paying close attention to modifications in their effects on behavior.

## 1. Introduction

The central nervous system (CNS), which comprises the brain and spinal cord, regulates vital processes, including cognition, motion, and emotion [1,2,3]. Neurological conditions such as Alzheimer’s disease (AD), Parkinson’s disease (PD), epilepsy, stroke, brain tumors, and psychiatric conditions, including major depressive disorders and schizophrenia, are examples of disorders that can affect the CNS [4,5,6]. To treat these conditions, medications must reach the cells and tissues of interest in the CNS. However, this is challenging because the blood–brain barrier (BBB) protects the CNS [7,8,9]. In addition to endothelial cells that are interconnected via junctional proteins, the BBB comprises structurally and functionally supporting cells, including astrocytes, pericytes, and microglia. The BBB is a natural defense mechanism that prevents toxins, pathogens, and foreign substances from entering the brain, where they can potentially cause harm [10,11,12]. However, it also restricts the delivery of most therapeutic agents because only small, lipophilic, and uncharged molecules can passively diffuse across the BBB [13,14,15]. Consequently, because it reduces the bioavailability and efficacy of numerous drugs, the BBB is a significant barrier to drug delivery to the brain.

The BBB restricts the passage of highly polar molecules, such as sugars, amino acids, peptides, and nucleosides, isolating the brain from many essential compounds [16,17,18,19]. Various modifications of their side chains have been explored to facilitate the penetration of these impermeable molecules [16,20,21,22]. One strategy involves the use of hydrocarbon “staples” to link the side chains of polar molecules, and the other is the use of N-methyl phenylalanine-rich peptides, which have been investigated as highly versatile BBB shuttles [23,24,25]. These modifications aim to enhance the ability of highly polar molecules to traverse the BBB, thereby enabling their access to the CNS for potential therapeutic and research applications. Modifying the polarity of molecules by altering their side chains can be an effective approach for enhancing their BBB permeability and improving their CNS efficacy [20,26,27,28].

**Figure 1:**
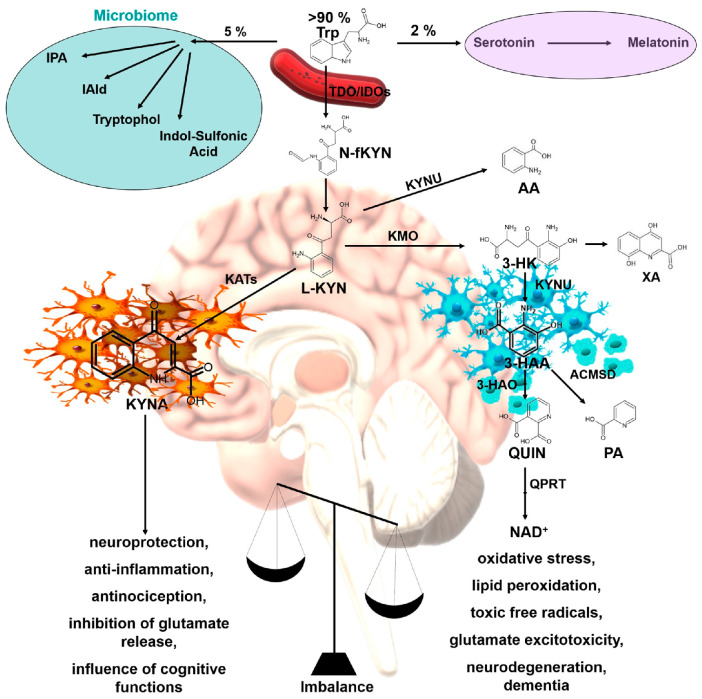
Tryptophan–kynurenine metabolic pathways and consequences of its imbalance. AA: anthranilic acid; ACMSD: 2-amino-3-carboxymuconate-6-semialdehyde decarboxylase; 3-HAA: 3-hydroxyanthranilic acid; 3-HAO: 3-hydroxyanthranilate oxidase; 3-HK: 3-hydroxy-L-kynurenine; IDOs: indoleamine 2,3-dioxygenases; KATs: kynurenine aminotransferases; KMO: kynurenine 3-monooxygenase; KYNA: kynurenic acid; KYNU: kynureninase; L-KYN: L-kynurenine; NAD^+^: nicotinamide adenine dinucleotide; N-fKYN: N-formyl-kynurenine; PA: picolinic acid; QPRT: quinolinate phosphoribosyltransferase; QUIN: quinolinic acid; TDO: tryptophan 2,3-dioxygenase; Trp: tryptophan; XA: xanthurenic acid; IAld: indole-3-aldehyde; IPA: indole-3-propionic acid.

Tryptophan (Trp) is an essential amino acid involved in various biological processes, such as the synthesis of protein, neurohormones such as serotonin and melatonin, and various indole derivatives [29,30,31,32,33]. The majority, exceeding 90%, of Trp within the human body undergoes metabolism via the kynurenine (KYN) pathway. This metabolic pathway is responsible for the production of various bioactive metabolites that exhibit a wide range of effects on both the central nervous and immune systems [34,35,36,37]. Dysregulation of the KYN metabolism has been implicated in various neuropsychiatric disorders, such as depression, schizophrenia, AD, and PD [38,39,40,41,42,43]. The degradation of KYNs occurs via two primary metabolic pathways: the neurotoxic pathway, which generates prooxidant molecules, and the neuroprotective pathway, which generates antioxidant molecules [44,45,46]. The maintenance of homeostasis and function in the CNS relies heavily on the equilibrium between various metabolic branches. Consequently, any disruption in this balance can result in the development of pathological conditions [47,48,49]. However, this balance can be disturbed by various factors, resulting in the accumulation of neurotoxic KYNs and the depletion of neuroprotective KYNs in the CNS [50,51,52]. This imbalance can cause neuronal damage, synaptic dysfunction, and cognitive impairment and contribute to the pathogenesis of neuropsychiatric disorders [53,54,55]. Therefore, KYN metabolism is a potential therapeutic target for neuropsychiatric disorders (Figure 1) [56,57,58].

Kynurenic acid (KYNA) is metabolized by the Trp-KYN metabolic pathway and functions as a neuroprotective metabolite [59,60,61]. Its antioxidant properties and antagonistic activity against ionotropic glutamate receptors, including N-methyl-D-aspartate (NMDA) receptors, are responsible for its neuroprotective effects [62,63,64]. KYNA has been implicated in various neuropsychiatric and neurodegenerative disorders, and KYNA levels in the brain and body are influenced by factors such as inflammation, stress, aging, and genetic variation [62,65,66,67,68,69]. Furthermore, recent research has shed light on the mechanism of emotional learning, including its potential role in modulating affective motor function and emotional responses [70,71,72,73,74,75,76,77]. The neural substrates involved in emotional learning, particularly KYNA, suggest a plausible impact on the limbic system, including structures such as the amygdala and prefrontal cortex, which are known to be involved in emotional regulation and associative learning [74,78,79,80,81,82,83,84,85,86]. For example, it has been shown that KYNA and its synthetic analogs, such as SZR-72 and SZR-104, possess the ability not only to influence motor domains of behavior but also to potentially modulate emotional responses [87]. Therefore, KYNA appears to be a potential drug candidate for the treatment of neuropsychiatric disorders because it can regulate the balance between neurotoxicity and neuroprotection [70,88,89,90,91,92,93,94,95,96].

**Figure 2:**
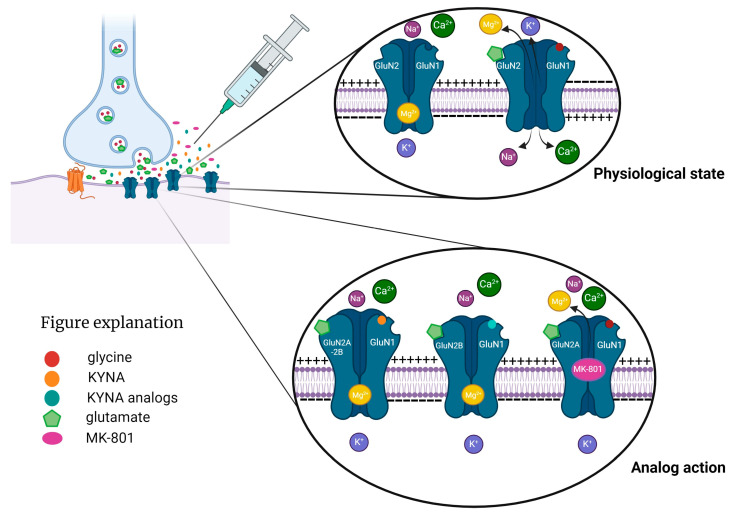
N-methyl-D-aspartic acid (NMDA) receptor complex physiological function in post- and extra-synaptic membranes of glutamatergic neurons and their function under the antagonistic effects of MK-801, kynurenic acid (KYNA), and KYNA analogs. NMDA receptors are made of four subunits: GLUNR1 and GLUNR3, which bind L-glycine and D-serine, and GLUNR2A and GLUNR2B, which bind glutamate. GLUNR2A supports cell survival, while GLUNR2B triggers cell death by allowing Ca^2+^ and Na^+^ influx. These subunits form a cationic channel that opens when L-glycine, D-serine, and glutamate bind simultaneously and Mg^2+^ is removed. NMDA receptor agonists with GLUNR2A may have therapeutic benefits. Created with BioRender.com.

However, more research is needed to evaluate its safety and efficacy and to consider its interactions with other metabolic pathways of Trp. KYNA binds to the receptor strychnine-insensitive glycine-binding site of the NMDA receptor [97,98]. At millimolar concentrations, KYNA inhibits the postsynaptic ionotropic glutamate receptor, the α-amino-3-hydroxy-5-methyl-4-isoxazolepropionic acid receptor, the kainate receptor, and interaction with G protein-coupled receptor 35 [99,100]. NMDA receptors are present in the mammalian brain in the post- and extra-synaptic membranes of glutamatergic neurons (Figure 2) [101,102]. We understand the various NMDA receptor subtypes associated with the gamma-aminobutyric acidergic and dopaminergic systems, as well as their functions [103,104,105,106]. 

**Figure 3:**
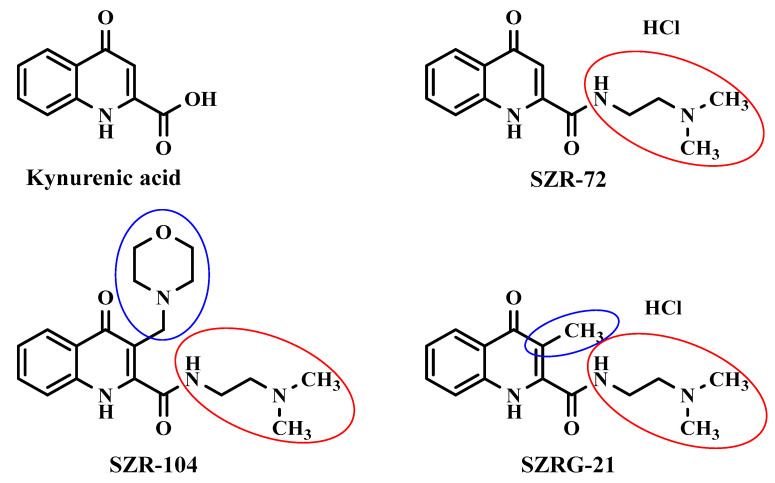
Chemical structures and highlighted differences in kynurenic acid and its analogs. SZR-72: *N*-(2-(dimethylamino)ethyl)-4-oxo-1,4-dihydroquinoline-2-carboxamide hydrochloride; SZR-104: *N*-(2-(dimethylamino)ethyl)-3-(morpholinomethyl)-4-oxo-1,4-dihydroquinoline-2-carboxamide; and SZRG-21: *N*-(2-(dimethylamino)ethyl)-3-methyl-4-oxo-1,4-dihydroquinoline-2-carboxamide hydrochloride.

The synthetic analogs of KYNA investigated in this study include *N*-(2-(dimethylamino)ethyl)-4-oxo-1,4-dihydroquinoline-2-carboxamide (SZR-72) as a KYNA amide derivative; *N*-(2-(dimethylamino)ethyl)-3-(morpholinomethyl)-4-oxo-1,4-dihydroquinoline-2-carboxamide (SZR-104) as an aminoalkylated amide derivative with the new function at C–3 position; and *N*-(2-(dimethylamino)ethyl)-3-methyl-4-oxo-1,4-dihydroquinoline-2-carboxamide (SZRG-21) that contains an alkyl group in the C–3 position as a transitional derivative between SZR-72 and SZR-104 (Figure 3). They are able to mimic the pharmacological actions of KYNA, including the antagonistic effects on glutamate receptors [107]. They can also affect the morphology and function of microglia, which are brain immune cells, as well as the expression and methylation of histone H3, a protein that controls gene transcription [107,108]. These analogs may have potential therapeutic applications for neuroinflammatory and neurodegenerative disorders.

Given their ability to mimic KYNA’s pharmacological actions, particularly its antagonistic effects on glutamate receptors, these analogs have the potential to alter the delicate balance within neural circuits implicated in conditions such as schizophrenia, bipolar disorder, and major depressive disorder, all of which are associated with glutamatergic dysregulation [89,109,110,111]. Moreover, the influence of these analogs on microglial morphology and function, along with their impact on histone H3 expression and methylation, suggests their broader implications in neuroinflammatory processes mediated in a wide range of interactions [73,112,113]. By modulating microglial behavior and epigenetic regulation, these analogs may exert neuroprotective effects, potentially attenuating the progression of debilitating disorders [114,115,116,117]. The multifaceted actions of these analogs on both the immune response and epigenetic regulation highlight their promise as a novel class of compounds for addressing the complex pathophysiological mechanisms underlying various neuropsychiatric and neurologic conditions [118,119,120].

Preclinical research has made significant contributions to drug design and data collection, including unexpected side effects, by assessing the complex in vivo consequences of newly developed compounds that would otherwise be impossible to achieve in clinical studies. The main objective of this study was to investigate the effects of KYNA and its analogs on the motor domain of behavior in mice. This study aimed to synthesize KYNA analogs, SZR-72, SZR-104, and SZRG-21, with different side chain modifications that may affect their permeability to the BBB and administer them intracerebroventricularly (i.c.v.) at two different doses (0.04 and 0.1 μmol/4 μL) to mice (Figure 3) in order to obtain the most objective and immediate feedback about the effects of analog molecules in vivo on individual brain regions and, thus, on memory and motor functions. The study also aimed to measure the exploratory and affective motor functions and motor skills of these mice using the most widely used animal models, such as the OF and RR tests, and compare the behavioral outcomes of KYNA and its analogs. This study further opens avenues to analyze the possible mechanisms underlying their effects on the motor domain of behavior and to evaluate their potential as novel therapeutic agents for neuropsychiatric disorders involving motor impairments.

## 2. Results

### 2.1. Behavioral Tests

A pilot study was conducted to determine KYNA’s testing doses in comparison with MK-801. KYNA in a particular dose was used as a control and was compared with KYNA analogs in equivalent doses in subsequent behavioral tests to assess the effects of KYNA analogs.

#### 2.1.1. Pilot study

**Figure 4:**
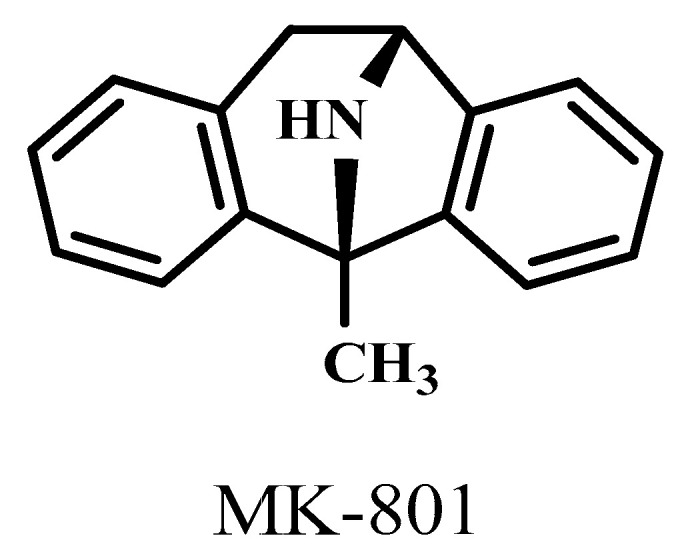
Chemical structure of MK-801.

**Figure 5:**
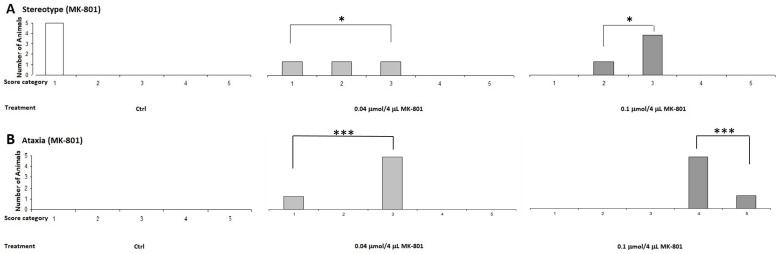
Effects of MK-801 on pilot stereotype/ataxia tests. MK-801 caused massive stereotypes and ataxia symptoms in mice after i.c.v. injection. (**A**) Stereotype symptom score for MK-801 treatment, 0.04 μmol/4 μL MK-801, vs. control (Ctrl) group, 0.1 μmol/4 μL MK-801 vs. Ctrl group (*p* = 0.017). (**B**) Ataxia symptoms score of MK-801 treatment, 0.04 μmol/4 μL MK-801, vs. Ctrl group, 0.1 μmol/4 μL MK-801 vs. Ctrl group (*p* = 0.001). We show the number of animals and the score categories. *: 0.05; ***: 0.001, N(Ctrl) = 5, N(0.04 μmol/4 μL MK-801) = 5, and N(0.1 μmol/4 μL MK-801) = 5.

The 1-methyl-8-azabicyclo[3.2.1]octane (Dizocilpine aka. MK-801) molecule (Figure 4) in a lower dose, 0.04 μmol/4 μL, caused ataxia symptoms in mice. However, KYNA did not cause any side effects at this dose (Figure 5).

**Figure 6:**
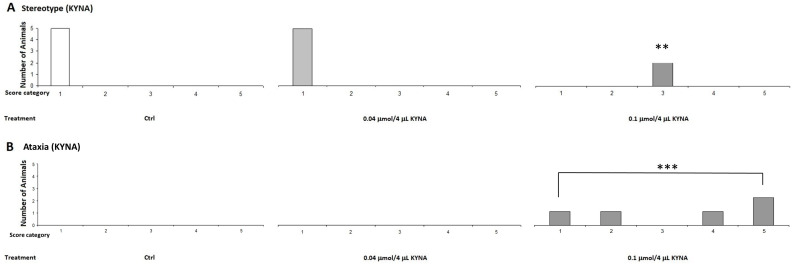
Effect of kynurenic acid (KYNA) on pilot stereotype/ataxia test. KYNA caused stereotype and ataxia symptoms at a higher dose after i.c.v. injection. (**A**) Stereotype symptom score of KYNA treatment, 0.1 μmol/4 μL KYNA, vs. control (Ctrl) group, 0.1 μmol/4 μL KYNA vs. 0.04 μmol/4 μL KYNA group (*p* = 0.004). (**B**) Ataxia symptoms score of KYNA treatment, 0.1 μmol/4 μL KYNA, vs. Ctrl group, 0.1 μmol/4 μL KYNA, vs. 0.04 μmol/4 μL KYNA group (*p* = 0.001). We show the number of animals and the score categories. **: 0.01; ***: 0.001, N(Ctrl) = 5, N(0.04 μmol/4 μL KYNA) = 5, and N(0.1 μmol/4 μL KYNA) = 5.

On the other hand, when the animals were treated with a higher dose, 0.1 μmol/4 μL, the ataxia and stereotype scores were higher in the MK-801-treated group than in the lower-dose group. We observed that the 0.1 μmol/4 μL dose induced ataxia symptoms in the KYNA group (Figure 6).

#### 2.1.2. Stereotype/Ataxia Test

**Figure 7:**
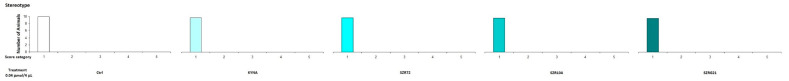
Effect of 0.04 μmol/4 μL doses of KYNA and its analogs in the stereotype and ataxia tests. Stereotype symptom scores after treatment were not significantly different between treatment groups. We show the number of animals and the score categories. N(Ctrl) = 10, N(0.04 μmol/4 μL KYNA) = 10, N(0.04 μmol/4 μL SZR-72) = 10, N(0.04 μmol/4 μL SZR-104) = 10, and N(0.04 μmol/4 μL SZRG-21) = 10.

**Figure 8:**
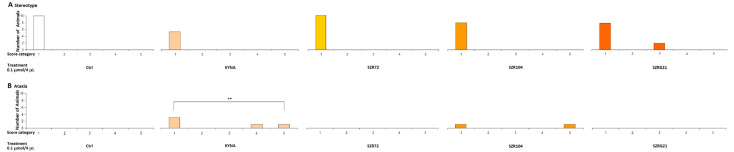
Effect of 0.1 μmol/4 μL doses of KYNA and its analogs in the stereotype and ataxia tests. (**A**) Stereotype symptom scores after treatment were not significantly different between treatment groups. (**B**) Ataxia symptom score after the treatment, 0.1 μmol/4 μL KYNA, vs. Ctrl, SZR-72, and SZRG-21 groups (*p* = 0.004). We show the number of animals and the score categories. N(Ctrl) = 10, N(0.1 μmol/4 μL KYNA) = 10, N(0.1 μmol/4 μL SZR-72) = 10, N(0.1 μmol/4 μL SZR-104) = 10, and N(0.1 μmol/4 μL SZRG-21) = 10.

In our previous experiments, we observed that MK-801 had a more pronounced effect at both doses, whereas the effect of KYNA was more pronounced at higher doses. Therefore, we sought to investigate the effects of KYNA and its analog molecules at both doses in further experiments. Specifically, we treated the right lateral brain ventricles of mice with KYNA, SZR-72, SZR-104, and SZRG-21 at 0.04 and 0.1 μmol/4 μL. We did not observe a significant difference in stereotype between the treated groups at the 0.04 μmol/4 μL dose (Figure 7), as this dose did not cause ataxia. However, at the 0.1 μmol/4 μL dose, we did observe a significantly higher ataxia score in the KYNA group (*p* = 0.004) than in the control, SZR-72, and SZRG-21 groups (Figure 8B), but we did not observe a significant difference in stereotype between the treated groups (Figure 8A).

#### 2.1.3. Open-Field (OF) Test

**Figure 9:**
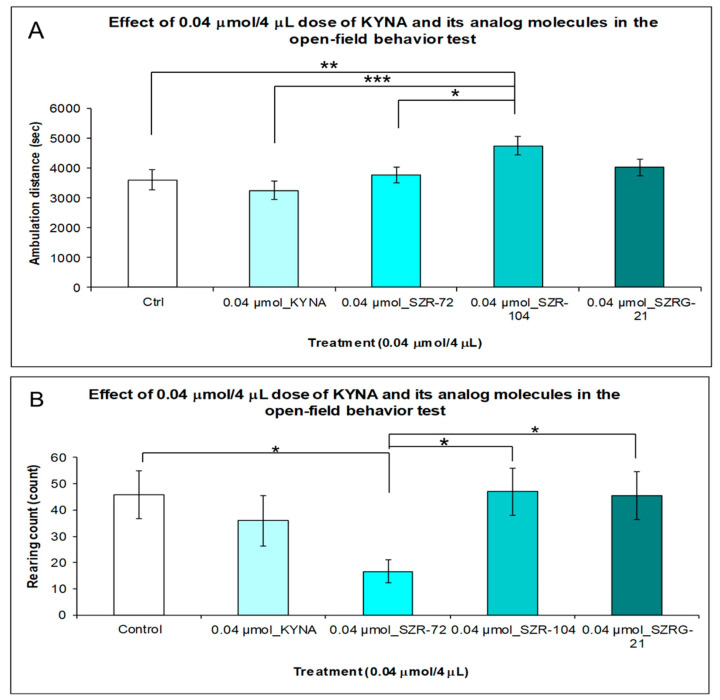
Open-field behavior test after treatment with KYNA and its analogs in 0.04 μmol/4 μL doses. (**A**) Ambulation distance: We found significant differences in behavior between the SZR-104-treated group and the control (Ctrl) (*p* = 0.01), KYNA (*p* = 0.001), or SZR-72 (*p* = 0.026) groups. (**B**) Rearing count: We found significant differences between the 0.04 μmol SZR-72 group vs. the Ctrl group (*p* = 0.019), vs. the SZR-104 group (*p* = 0.015), and vs. the SZRG-21 group (*p* = 0.02). We show the data mean ± SEM. *: 0.05; **: 0.01; ***: 0.001, N(Ctrl) = 10, N(0.04 μmol/4 μL KYNA) = 10, N(0.04 μmol/4 μL SZR-72) = 10, N(0.04 μmol/4 μL SZR-104) = 10, and N(0.04 μmol/4 μL SZRG-21) = 10.

Ten minutes after that we i.c.v. treated the animals with both doses (0.04 and 0.1 μmol/4 μL) of KYNA and its analog molecules. After the stereotype/ataxia behavior test was monitored, we observed the spontaneous locomotor and exploration activities of mice. The mice were inserted into the center of the open-field box, and their behavior was measured for 15 min. The results of the experiment showed a significant difference in the horizontal motion (ambulation distance) between the 0.04 μmol/ 4 μL dose of KYNA and the analog-treated groups. The ambulation distance was significantly higher in the mice treated with SZR-104 than in the control (*p* = 0.01), KYNA (*p* = 0.001), and SZR-72 (*p* = 0.026) groups (Figure 9A). The vertical motion (rearing count) was significantly different between the SZR-72 and control (*p* = 0.019), SZR-104 (*p* = 0.015), and SZRG-21 (*p* = 0.02) groups (Figure 9B).

**Figure 10:**
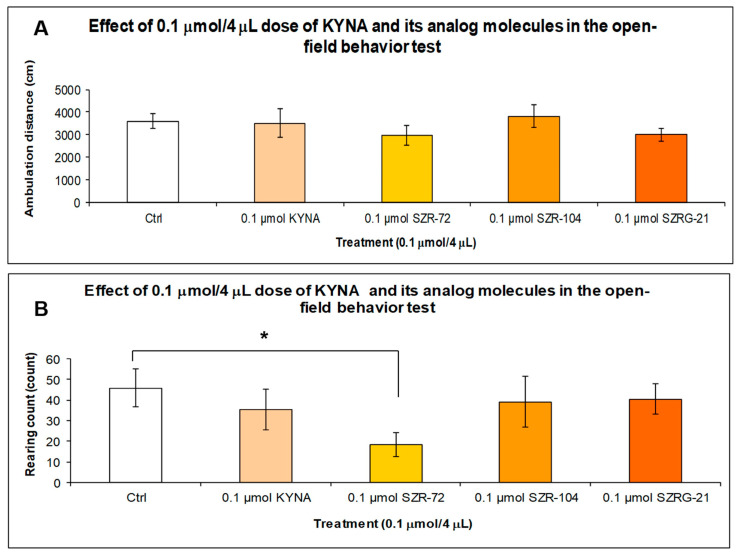
Open-field (OF) test after treatment with KYNA and its analogs in 0.1 μmol/4 μL doses. (**A**) Ambulation distance: We did not find significant differences in behavior between the treated groups and the control (Ctrl) group. (**B**) Rearing count: We found a significant difference only between the 0.1 μmol SZR-72 group vs. the Ctrl group (*p* = 0.04). We show the data mean ± SEM. *: 0.05, N(Ctrl) = 10, N(0.1 μmol/4 μL KYNA) = 10, N(0.1 μmol/4 μL SZR-72) = 10, N(0.1 μmol/4 μL SZR-104) = 10, and N(0.1 μmol/4 μL SZRG-21) = 10.

When we treated the animals with a 0.1 μmol/4 μL dose of KYNA and its analog molecules, there was no significant difference in horizontal motion (ambulation distance) between the treated groups (Figure 10A). However, in vertical motion (rearing count), there was a significant difference (*p* = 0.04) between the 0.1 μmol/4 μL dose of SZR-72 and the control group (Figure 10B).

#### 2.1.4. Rotarod (RR) Test

**Figure 11:**
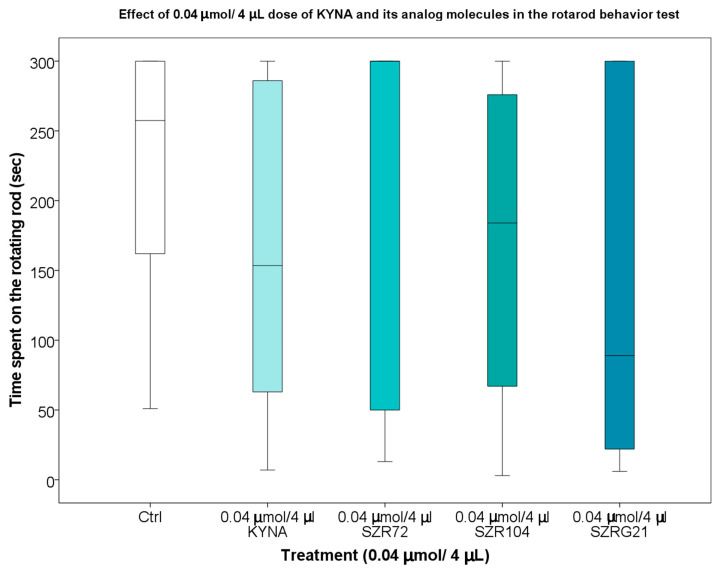
Rotarod behavior test of mice treated with 0.04 μmol/4 μL of KYNA and its analog molecules. We show the data median ± SD. N(Ctrl) = 10, N(0.04 μmol/4 μL KYNA) = 10, N(0.04 μmol/4 μL SZR-72) = 10, N(0.04 μmol/4 μL SZR-104) = 10, and N(0.04 μmol/4 μL SZRG-21) = 10.

**Figure 12:**
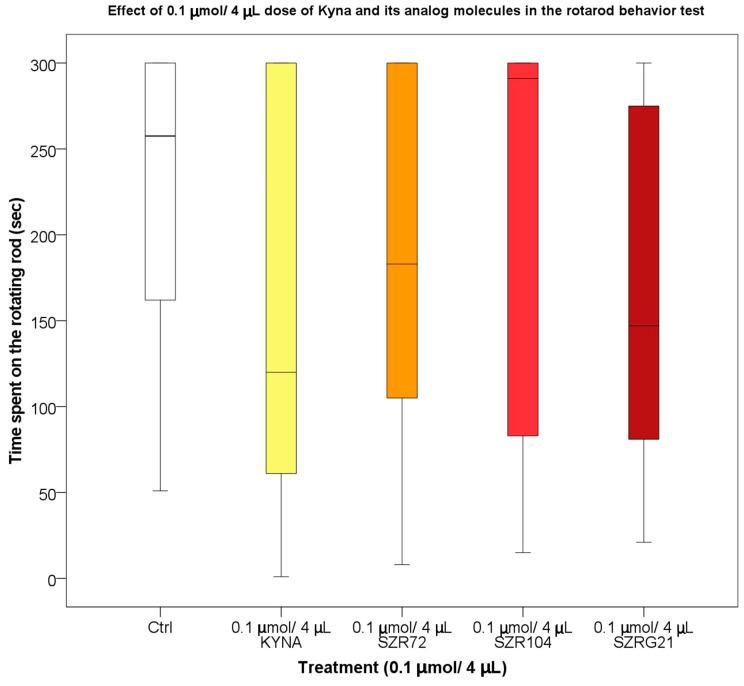
Rotarod behavior test of mice treated with 0.1 μmol/4 μL KYNA and its analog molecules. We show the data median ± SD. N(Ctrl) = 10, N(0.1 μmol/4 μL KYNA) = 10, N(0.1 μmol/4 μL SZR-72) = 10, N(0.1 μmol/4 μL SZR-104) = 10, and N(0.1 μmol/4 μL SZRG-21) = 10.

Based on data from previous experiments, it was confirmed that KYNA did not accumulate in the extracellular space; it eluted rapidly [121], and the KYNA concentration in the mouse serum and CNS samples decreased after 30–40 min [122]. Therefore, we were interested in the effect of KYNA on motor coordination and balance in mice 25–30 min after the i.c.v. treatment. During our investigations, we found that the 0.04 and 0.1 μmol/4 μL doses of KYNA and its analogs after i.c.v. injection did not significantly affect the locomotion skills of the mice (Figure 11 and Figure 12).

## 3. Discussion

The CNS plays a critical role in regulating essential functions such as cognition, motion, and emotion [123,124,125,126]. However, they are susceptible to various disorders that impair their function [4,5,6,127,128,129,130,131]. One of the major challenges in treating these disorders is the BBB, a semi-permeable membrane that protects the CNS from harmful substances but also limits the delivery of therapeutic agents to the brain [15,132,133,134]. The BBB is composed of various cells, junctions, and transporters that regulate the transport of molecules between the blood and brain [135,136,137,138]. Strategies have been developed to overcome this barrier by modifying the polarity of highly polar molecules such as sugars, amino acids, peptides, and nucleosides, which are essential for the CNS but cannot cross the BBB [139,140,141]. These modifications enhance the BBB permeability of these molecules, enabling their access to the CNS for potential therapeutic and research applications [20,142,143,144].

Trp is metabolized into neurotoxic and neuroprotective KYNs [44,45,46]. The balance of these KYNs affects the CNS and the immune system, and its dysregulation is linked to neuropsychiatric disorders [38,39,40,145,146,147]. Neurotoxic and neuroprotective branches of KYNs, the equilibrium of which is critical for maintaining homeostasis and function in the CNS, have been implicated in its degradation [47,48,49,148,149]. Conversely, KYN metabolites demonstrate an extensive array of bioactive characteristics, including but not limited to immunomodulating, oxidant, antioxidant, anti-inflammatory, and neurotoxin actions [150,151,152]. The specific effects of these metabolites are contingent upon their concentration and the cellular milieu [35,153,154]. Furthermore, the metabolic system operates within intricate positive and negative feedback loops [66,155,156,157]. Furthermore, a critical aspect is the absence of consensus concerning the functionalities of KYN metabolites.

KYNA is a neuroprotective KYN that possesses antioxidant properties, antagonizes glutamate receptors, and modulates immunity and digestion [158,159,160]. KYNA is involved in neurological disorders, is affected by various factors, and is a potential drug candidate for neuropsychiatric conditions [91,161,162,163,164]. The KYNA analog SZR-72 has been demonstrated to attenuate the severity of acute necrotizing pancreatitis in experimental settings, regulate body weight and home-cage activity in mice, and inhibit nitroglycerol-induced enhancement in c-Fos immunoreactivity within the rat caudal trigeminal nucleus [165,166,167]. Its effects are comparable to those of KYNA. Similarly, SZR-104 has been shown to modulate immune systems and inhibit glutamate receptors with effects similar to KYNA [168]. In animal models, SZR-104 has been found to inhibit epileptiform seizures [169]. Furthermore, SZR-104 alters the intracellular distribution and methylation patterns of histone H3, a protein that regulates gene expression [108]. These findings indicate the potential therapeutic applications of these compounds in neurological and psychiatric disorders. However, further studies are required to evaluate its safety and efficacy. SZRG-21, the C-3 alkyl group-transitional derivative of SZR-72 and SZR-104, is a recently synthesized analog whose biological functions have yet to be investigated.

Firstly, this pilot study showed the effects of KYNA and MK-801, two NMDA receptor antagonists, on mice behavior. MK-801 caused more ataxia and stereotypes than KYNA at both doses (0.04 and 0.1 μmol/4 μL). KYNA causes ataxia at a higher dose. Our previous study showed that KYNA elicits antidepressant-like effects and improves learning and memory [163,170]. We investigated the effects of KYNA and its analogs (SZR-72, SZR-104, and SZRG-21) on the spontaneous locomotor and exploratory activities of mice after i.c.v. injections. The horizontal and vertical motions of the mice were measured in an OF box for 15 min. The lower dose (0.04 μmol/4 μL) of SZR-104 increased horizontal motion, while the lower dose of SZR-72 decreased vertical motion compared with the control and other groups. The higher dose (0.1 μmol/4 μL) of SZR-72 decreased vertical motion compared with the control group. The other groups did not show any significant differences at the higher doses. It was also observed that KYNA and its analogs did not affect the motor coordination and balance of mice 25–30 min after i.c.v. injections.

Studying the effects of MK-801, KYNA, and KYNA analogs on mouse behavior presents several challenges and requires specific knowledge and technology. This research found that MK-801 caused more ataxia and stereotypes than KYNA at both doses, whereas KYNA caused ataxia at a higher dose. Additionally, the study tested KYNA analogs and found that they did not cause significant behavioral changes. To achieve these results, researchers need a deeper understanding of NMDA receptor antagonists, mouse behavior, and the specific effects of KYNA and its analogs. The technology required for this study includes precise dosing and administration methods for i.c.v. injections, as well as behavioral testing equipment to measure ataxia, stereotypes, and spontaneous locomotor and exploration activities in mice [171,172,173]. Additional research is warranted in this field, as these results supplement current knowledge showing that specific dosages of KYNA enhance cognition and memory in addition to its antidepressant-like properties and pain modulation [163,170,174]. Additionally, KYNA analogs have exhibited promising results in animal models of various neurological and psychiatric disorders, although their mechanisms of action, pharmacokinetics, and safety warrant further investigation [165,166,167,168,175]. Therefore, KYNA analogs represent a novel class of drugs with the potential for future clinical applications. The limitations of this study lie in the fact that in vitro studies have not revealed the precise action sites and mechanisms of KYNA analogs. Beyond the molecular level, there could be a higher order of interaction. Multiple orders of behavior make it difficult to determine the precise mechanisms involved in the modification of behavior. This study demonstrated the action of KYNA analogs via i.c.v. administration; however, it does not imply that peripheral administration will produce similar results.

Preclinical research, including in vitro and in vivo studies, can provide invaluable data that are not feasible to investigate in humans [176,177,178,179,180,181,182,183,184,185,186,187,188,189,190]. This study showed that the incorporation of the C-3 side chain elicits subtle differences in curiosity and emotion in animal models of motor function. Ongoing clinical studies have advanced our understanding of the behavioral domains in neuropsychiatric conditions and have sought their potential management [184,191,192,193,194,195,196,197,198,199]. Furthermore, computational strategies accelerate our advancement in comprehending their pathology, as well as in developing novel strategies for the management of neurological and psychiatric disorders, including neurotropic computer-assisted drug design [200,201,202,203,204,205,206,207,208]. Integrating these interdisciplinary approaches further adds impetus and optimizes strategies, including drug development research, leading to the testing and assessment of potential lead compounds. These outcomes enable researchers to evaluate the effects of novel interventional approaches such as drug-assisted brain stimulation [106,209,210,211,212,213,214]. These methods demonstrate promise for the development of new and more effective treatments, including novel drugs [215]. Furthermore, advanced imaging techniques have played a significant role in brain research [216,217,218]. Neuroimaging studies have uncovered structural and functional brain changes that are associated with neuropsychiatric disorders and therapeutics [219,220,221,222]. These imaging techniques can aid in identifying unique clinical cases and provide valuable insights into the pathophysiology of these disorders and novel therapeutic strategies [214,223,224,225]. Further research is needed to determine the optimal concentration for neuroprotection and the threshold for neurotoxicity. The findings of this study suggest that KYNA analogs represent a new class of drugs with potential clinical applications in neurological and psychiatric disorders.

## 4. Materials and Methods

### 4.1. Materials

We utilized a solution containing 0.9% saline (B. Braun Melsungen AG, Hessen, Germany) in a 4 μL volume, as well as MK-801 (Sigma-Aldrich Ltd., Budapest, Hungary) and KYNA (Sigma-Aldrich Ltd., Budapest, Hungary) molecules in a pilot study. In our experiment, we employed 0.9% saline and KYNA (Sigma-Aldrich Ltd., Budapest, Hungary) in a 0.1 μmol/4 μL dose, as well as KYNA analogs (SZR-72, SZR-104, SZRG-21) in equal doses to KYNA. The new analogs were synthesized at the Faculty of Pharmacy, Institute of Pharmaceutical Chemistry, University of Szeged, using the procedures described in Section 4.2. Fresh solutions of MK-801 and KYNA and its analogs were prepared by dissolving them in 0.9% aqueous saline and adjusting the pH to 7.4.

### 4.2. Kynurenic Acid Analog Synthesis

**Figure 13:**
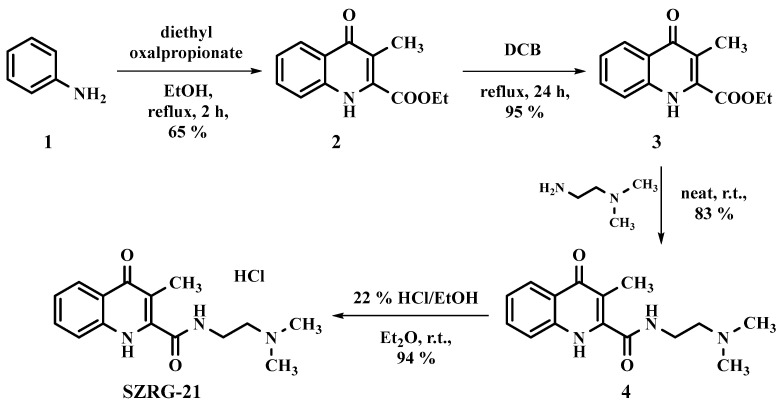
Synthesis of SZRG-21 (N-(2-(dimethylamino)ethyl)-3-methyl-4-oxo-1,4-dihydroquinoline-2-carboxamide).

The synthesis of KYNA analogs SZR-72 and SZR-104 was carried out in accordance with previously reported methods [175]. The synthesis of compound SZRG-21 commenced with aniline **1** (1.00 g, 10.70 mmol), which was reacted with diethyl oxalpropionate (4 mL, ρ = 1.073 g/mL, 21.2 mmol) in ethanol (20 mL) under reflux conditions. The progress of the reaction was monitored by TLC (eluent = n-hexane:ethyl acetate, 4:1), and once the reaction was complete, it was cooled to room temperature. Fewer side products were observed compared with the reaction run in acetic acid, which is commonly used as a solvent in the literature for synthesizing this compound [226,227,228,229]. After evaporation of the ethanol under reduced pressure, column chromatography was employed for purification using n-hexane:ethyl acetate (4:1) as the eluent. Diethyl 2-methyl-3-(phenylamino)maleate (**2**) was obtained as a yellow oil: yield = 0.81 g (65 %; Figure 13).

^1^H (500 MHz, DMSO), δ(ppm): 1.06 (3H, t, *J* = 7.2 Hz); 1.25 (3H, t, *J* = 6.9 Hz); 1.77 (3H, s); 4.12–4.21 (4H, m); 6.99 (2H, d, *J* = 7.2 Hz); 7.07 (1H, t, *J* = 7.5 Hz); 7.30 (t, 2H, *J* = 7.6 Hz, 1H); 10.01 (1H, s), ^13^C (125 MHz, DMSO), δ(ppm): 13.6, 13.9, 14.7, 60.4, 62.2, 95.9, 120.7, 124.2, 129.8, 140.5, 146.9, 164.7, 169.9 (Figures S1 and S2).

The solvents used during the ring closure process were modified from those reported in the literature, including PPA, mineral oil, and diphenyl ether; instead, 1,2-dichlorobenzene (DCB, 20 mL) was employed as the solvent. The reaction was carried out at reflux temperature for a total of 24 h, resulting in almost complete conversion of the starting material into compound **3**, with only minor side products evident (as determined by TLC with the eluent DCM:MeOH 19:1). After the removal of the solvent under reduced pressure, crystallization was induced using Et_2_O (3 mL), yielding a beige crystal with a mass of 0.64 g (95%; Figure 13).

The amidation process was carried out under neat conditions, starting with 3 (0.60 g, 2.59 mmol) and employing an excess of *N*^1^,*N*^1^-dimethylethane-1,2-diamine at room temperature. The progress of the reaction was assessed by TLC (eluent: DCM:MeOH 19:1), and upon completion, 5 mL of DCM was added. The resulting precipitate was filtered and washed with diethyl ether in two separate instances (2 × 10 mL). The final product (**4**) was a white crystal, yield = 0.59 g (83%; Figure 13), exhibiting a melting point of 192–194 °C.

^1^H (500 MHz, DMSO), δ(ppm): 1.99 (3H, s); 2.20 (6H, s); 2.43 (2H, t, *J* = 6.5 Hz); 3.33–3.43 (2H, m); 7.29 (1H, t, *J* = 7.5 Hz); 7.57–7.65 (2H, m); 8.08 (1H, d, *J* = 7.6 Hz); 8.72–8.79 (1H, m); ^13^C (125 MHz, DMSO), δ(ppm): 11.7, 37.7, 45.7, 58.3, 113.5, 118.7, 123.3, 123.8, 125.4, 132.0, 139.6, 143.6, 163.9, 187.7 (Figures S3 and S4).

SZRG-21 was prepared by initiating from **4** (0.55 g, 2.01 mmol) in Et_2_O (10 mL). HCl/EtOH (22%) was then slowly added until the pH reached 1–2. The resulting crystals were filtered and washed with Et_2_O (2 × 10 mL). The yield of SZRG-21 was 0.59 g (94 %). During melting point determination, SZRG-21 decomposed at 300 °C (Figure 13).

^1^H (500 MHz, DMSO), δ(ppm): 2.00 (3H, s); 2.85 (6H, d, *J* = 4.8 Hz); 3.31 (2H, q, *J* = 6.1 Hz); 3.66 (2H, q, *J* = 5.9 Hz), 7.32 (1H, t, *J* = 7.6 Hz); 7.66 (1H, t, *J* = 7.2 Hz); 7.77 (1H, d, *J* = 8.1 Hz); 8.11 (1H, d, *J* = 7.8 Hz); 9.18 (1H, t, *J* = 5.5 Hz); 10.43 (1H, brs); 12.45 (1H, brs); ^13^C (125 MHz, DMSO), δ(ppm): 11.6, 34.8, 42.8, 55.7, 113.9, 118.8, 123.6, 123.7, 125.3, 132.2, 139.5, 142.8, 164.3 (Figures S5 and S6).

### 4.3. Animals

The study utilized male C57BL6/J mice (Mus musculus, Charles River Laboratories, Erkrath, Germany) that weighed between 25 and 30 g. These animals were 10–12 weeks old and housed in cages containing a maximum of five mice per cage. The mice were kept under standard laboratory conditions, including access to tap water and regular mouse chow, five animals per cage, and were maintained on a 12 h light–dark cycle at a temperature of 24 ± 1 °C and a humidity of 50 ± 10%. The animals were handled in accordance with the Regulations of the Faculty of Medicine, University of Szeged, Ethical Committee for the Protection of Animals in Research. This study was approved by the Ethical Committee for the Protection of Animals in Research at the University of Szeged (XI/275/2023), the Hungarian Health Committee (40/2013 (II.14.)), and European Community Council Directive 2010/63/EU.

### 4.4. Surgery

Mice were anesthetized with 4% chloral hydrate (Sigma-Aldrich Ltd., Budapest, Hungary) at a dose of 0.4 g/kg body weight. A polyethylene cannula (Fisher Scientific, Intramedic Clay Adams polyethylene tube, Budapest, Hungary) was inserted into the right lateral brain ventricle and fixed to the skull using cyanoacrylate (Ferrobond, Budapest, Hungary). The stereotaxic coordinates were set at anterior–posterior 0.2 mm, medial–lateral 1.09 mm to the bregma, with the cannula extending 2.3 mm deep into the skull surface. After a recovery period of five days, the mice were used for the experiments. On the 8th day following surgery, KYNA, its analogs, or saline (4 µL) were injected into the right lateral brain ventricle of the mice using an infusion pump (KD Scientific, Holliston, MA, USA) at a rate of 8 µL/min. The correct location of the inner i.c.v. cannula was confirmed by injecting 1% methylene blue solution after the experiment [230].

### 4.5. Behavioral Tests

The tests were carried out at the same time of the day to minimize variations in the diurnal rhythm of the animals. Prior to assessing each animal, the equipment was thoroughly cleaned with 70% alcohol to remove lingering scents [231,232]. We used five mice per group in the pilot study and ten mice per group in subsequent tests.

#### 4.5.1. Pilot Study

To determine the most effective dose of KYNA that did not cause severe side effects, we compared KYNA with an NMDA receptor antagonist molecule, MK-801. We treated the animals with 0.9% saline in 4 μL volumes of MK-801 in 0.04 and 0.1 μmol/4 μL doses and KYNA in equal doses to MK-801 (Figure 4). These molecules were administered to mice in the right lateral brain ventricle. We observed the effect of MK-801 and KYNA using the stereotype/ataxia behavior test after i.c.v. injections. The duration of the observation was 10 min.

#### 4.5.2. Stereotype Behavior and Ataxia

Behavioral changes, including ataxia and stereotyped behavior, were observed and recorded for 10 min after treatment. The mice were then monitored for an additional 10 min, during which, their behavior was rated using the scale described by Sturgeon et al. (1979) [233] and Contreras (1990) [234]. The scale measures five categories of stereotyped behavior, including sniffing, grooming, and rearing behavior, as well as reciprocal forepaw treading or undirected head movement, backward walking, head weaving, circling behavior, continuous head weaving, circling, or backward walking and dyskinetic extensions or flexion of the limbs, head, and neck or weaving greater than four. For ataxia, the scale measures awkward and jerky movements; stumbling or awkward posture; falling; inability to move beyond a small area or support weight on the stomach or haunches; and inability to move, except for twitching movements.

#### 4.5.3. Open-Field (OF) Test

Spontaneous locomotor and exploratory activities were assessed using an automated tracking system within the activity chamber. The chamber was linked to a computer that recorded the exploration and locomotor activity of the subjects [235,236,237]. Each animal was placed individually at the center of a black box measuring 60 cm × 60 cm × 70 cm, which was equipped with automated infrared photocells for precise measurements. The box platform was divided into nine equal squares. The animals were allowed to move freely for 15 min and divided into three distinct sessions. The movement signals were analyzed using Conducta 1.0. We quantified ambulation distance and the duration of rearing, which serve as indicators of horizontal and vertical movement, spontaneous locomotor activity, and exploratory behavior.

#### 4.5.4. Rotarod (RR) Test

The rotarod test was used to evaluate motor coordination and balance. The rod, which rotated around its longitudinal axis, was positioned horizontally, and the animals were required to walk forward to maintain equilibrium and prevent falling off. The time taken for the mice to fall from the rotating rod was measured using an infrared sensor, and the latency to fall was automatically scored [238,239]. Motor coordination was assessed by comparing the latency to fall between the treatment groups, whereas motor learning was evaluated by comparing the first trial to subsequent trials after training, which demonstrated an increase in latency to fall over time. Prior to training, each mouse was acclimatized to the device for one hour at rest. On the first and second days, the animals were trained at a constant speed of 5 revolutions per minute (rpm) on a rotating rod, with three trials lasting 5 min each and 15 min intervals between trials. On the third day, 25 min after intraventricular (i.c.v.) injection, the latency at which each mouse fell off the rod in standard mode (5 rpm within 5 min) was recorded.

### 4.6. Statistical Analysis

In our study, we utilized Microsoft SPSS software (version 2.0) for the statistical analysis of our data. To evaluate our results, we employed a one-way ANOVA test that was adjusted using both the LSD and Bonferroni post hoc tests (*p* < 0.05), as well as the Kruskal–Wallis non-parametric test.

## 5. Conclusions

The BBB hinders drug delivery to the CNS, where many neuropsychiatric disorders arise. KYNA, a Trp metabolite, regulates CNS functions such as cognition, mood, and motor activity. Abnormal KYNA levels are linked to disorders such as schizophrenia, depression, and AD. However, KYNA cannot cross the BBB owing to its polarity. In this study, we prepared three KYNA analogs with different side chains, SZRG-21, SZR-72, and SZR-104, to improve their lipophilicity and BBB permeability. We tested their effects on mice behavior using the OF and RR tests. We found that SZR-104 increased horizontal exploration, whereas SZR-72 decreased vertical motion. Neither KYNA nor its analogs affected motor skills. These results show that side chain modification alters KYNA’s behavioral effects and its interactions with its receptors in the CNS. The present study provides novel perspectives on the pharmacological characteristics of KYNA-based medications for the treatment of neuropsychiatric disorders, as well as their therapeutic properties. This study emphasizes the importance of conducting further research, both in vitro and in silico, to explore the effects of KYNA and its analogs on the CNS.

## Data Availability

Data are contained within the article and Supplementary Materials.

## Supplementary Materials

The supporting information can be downloaded at https://www.mdpi.com/article/10.3390/ijms25063394/s1.

## Abbreviations

AA	anthranilic acid
ACMSD	2-amino-3-carboxymuconate-6-semialdehyde decarboxylase
AD	Alzheimer’s disease
BBB	blood–brain barrier
CNS	central nervous system
IDOs	indoleamine 2,3-dioxygenases
3-HAA	3-hydroxyanthranilic acid
3-HK	3-hydroxy-L-kynurenine
3-HAO	3-hydroxyanthranilate oxidase
KAT	kynurenine aminotransferase
KMO	kynurenine 3-monooxygenase
KYN	kynurenine
KYNA	kynurenic acid
KYNU	kynureninase
MK-801	1-methyl-8-azabicyclo[3.2.1]octane
NADH	nicotinamide adenine dinucleotide + hydrogen
N-fKYN	N-formyl-kynurenine
NMDA	N-methyl-D-aspartic acid
PA	picolinic acid
PD	Parkinson’s disease
QPRT	quinolinate phosphoribosyltransferase
QUIN	quinolinic acid
SZR-72	*N*-(2-(dimethylamino)ethyl)-4-oxo-1,4-dihydroquinoline-2-carboxamide
SZR-104	*N*-(2-(dimethylamino)ethyl)-3-(morpholinomethyl)-4-oxo-1,4-dihydroquinoline-2-carboxamide
SZRG-21	*N*-(2-(dimethylamino)ethyl)-3-methyl-4-oxo-1,4-dihydroquinoline-2-carboxamide
TDO	tryptophan 2,3-dioxygenase
Trp	tryptophan
XA	xanthurenic acid
