# Supplementary material for: The Impact of C-3 Side Chain Modifications on Kynurenic Acid: A Behavioral Analysis of Its Analogs in the Motor Domain
Source: Int J Mol Sci. 2024 Mar 16;25(6):3394. doi: 10.3390/ijms25063394 (PMC10970565; doi:10.3390/ijms25063394)
Supplement: Supplementary file 1 [file ijms-25-03394-s001.zip › ijms-2901932-SM.pdf]

# Supplementary Information

## The Impact of C-3 Side Chain Modifications on Kynurenic Acid: A Behavioral Analysis of Its Analogs in the Motor Domain


**Authors**

Diána Martos [1], Bálint Lőrinczi [2], István Szatmári [2], László Vécsei [1,3,†,*] and Masaru Tanaka [1,†,*]

**Affiliations**

[1] HUN-REN-SZTE Neuroscience Research Group, Hungarian Research Network, University of Szeged Danube Neuroscience Research Laboratory, Tisza Lajos krt. 113, H-6725 Szeged, Hungary; martos.diana@med.u-szeged.hu (D.M.); vecsei.laszlo@med.u-szeged.hu (L.V.); tanaka.masaru.1@med.u-szeged.hu (M.T.)

[2] Institute of Pharmaceutical Chemistry and HUN-REN–SZTE Stereochemistry Research Group, University of Szeged, Eötvös u. 6, H-6720 Szeged, Hungary; lorinczi.balint@szte.hu (B.L.); szatmari.istvan@szte.hu (I.S.)

[3] Department of Neurology, Albert Szent-Györgyi Medical School, University of Szeged, Semmelweis u. 6, H-6725 Szeged, Hungary

* Correspondence: vecsei.laszlo@med.u-szeged.hu (L.V.); tanaka.masaru.1@med.u-szeged.hu (M.T.); Tel.: +36-62-545-351(L.V.); +36-62-342-847 (M.T.)

† These authors contributed equally to this work.


## Contents


1. $^{1}H$ NMR and $^{13}C$ NMR spectra of synthesized compounds

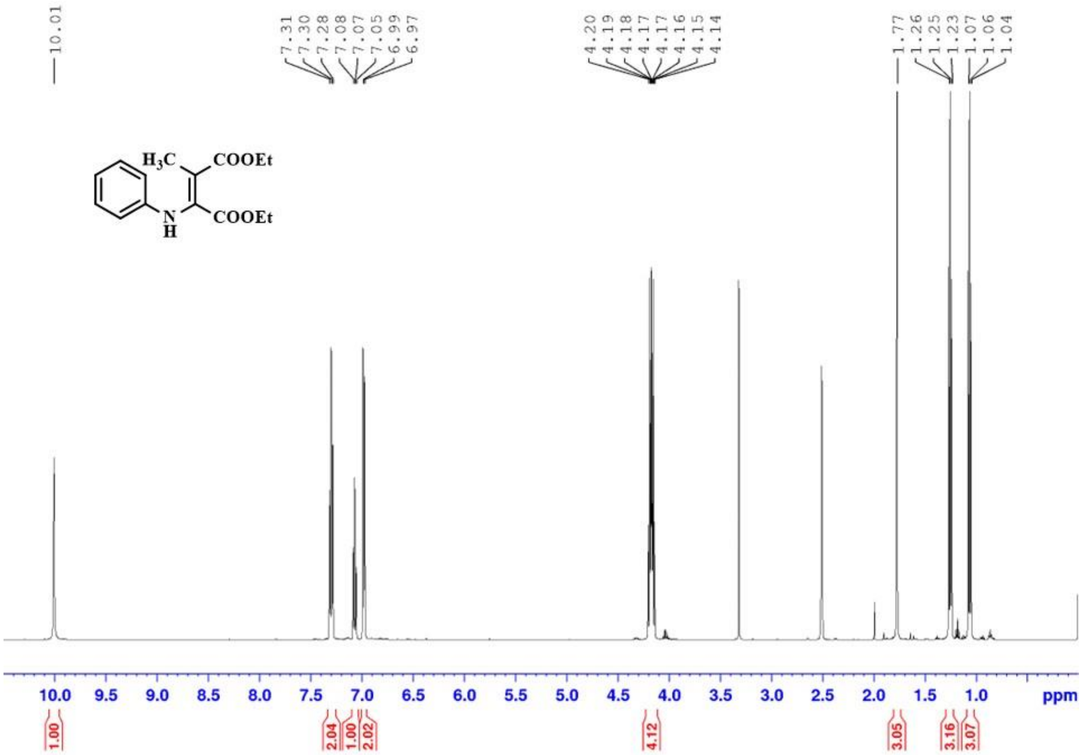


**Figure S1.** $^{1}$H-NMR spectrum of Diethyl 2-methyl-3-(phenylamino)maleate

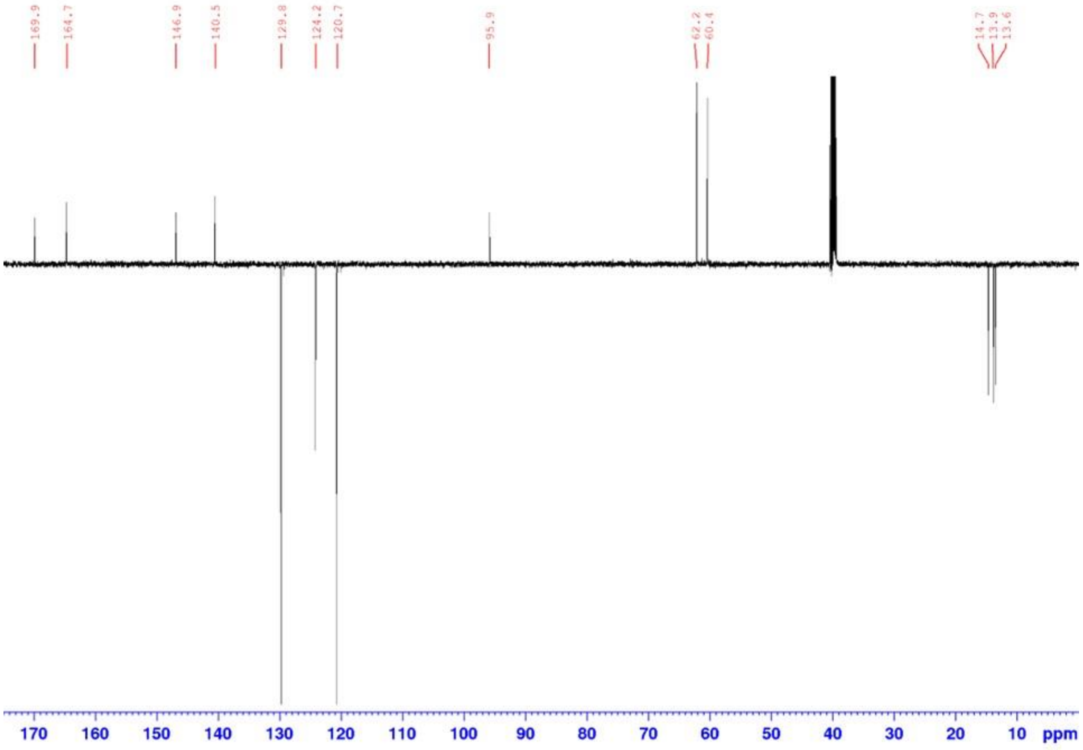


**Figure S2.** $^{13}$C-NMR spectrum of Diethyl 2-methyl-3-(phenylamino)maleate

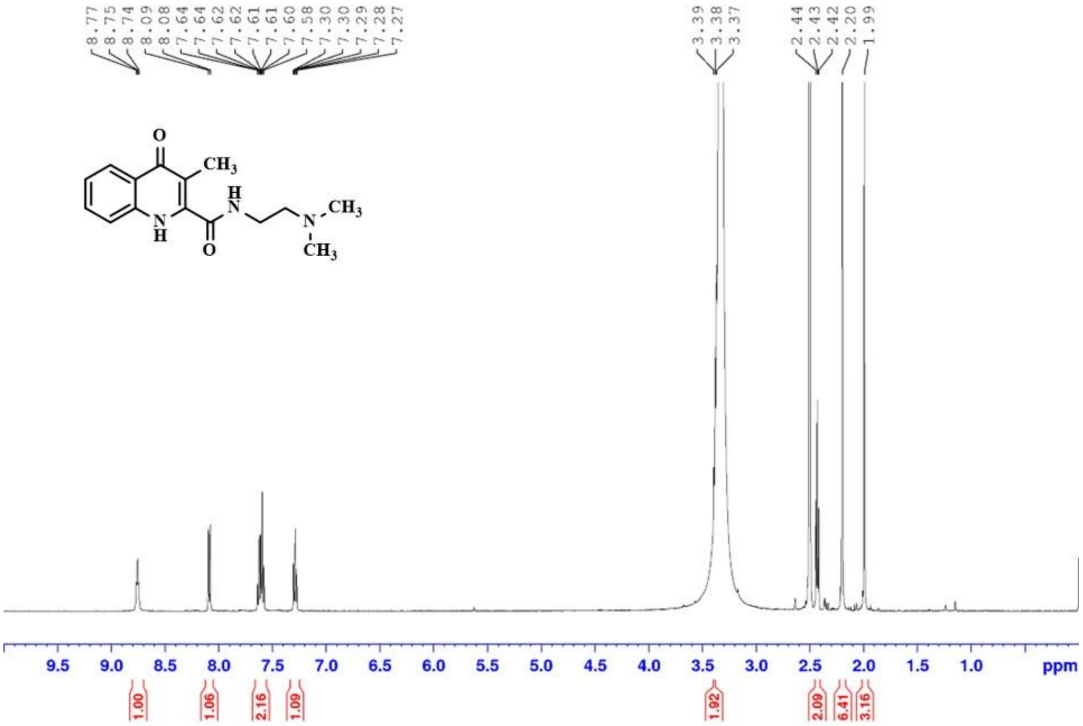


**Figure S3.** $^{1}$H-NMR spectrum of *N*-(2-(dimethylamino)ethyl)-3-methyl-4-oxo-1,4-dihydroquinoline-2-carboxamide

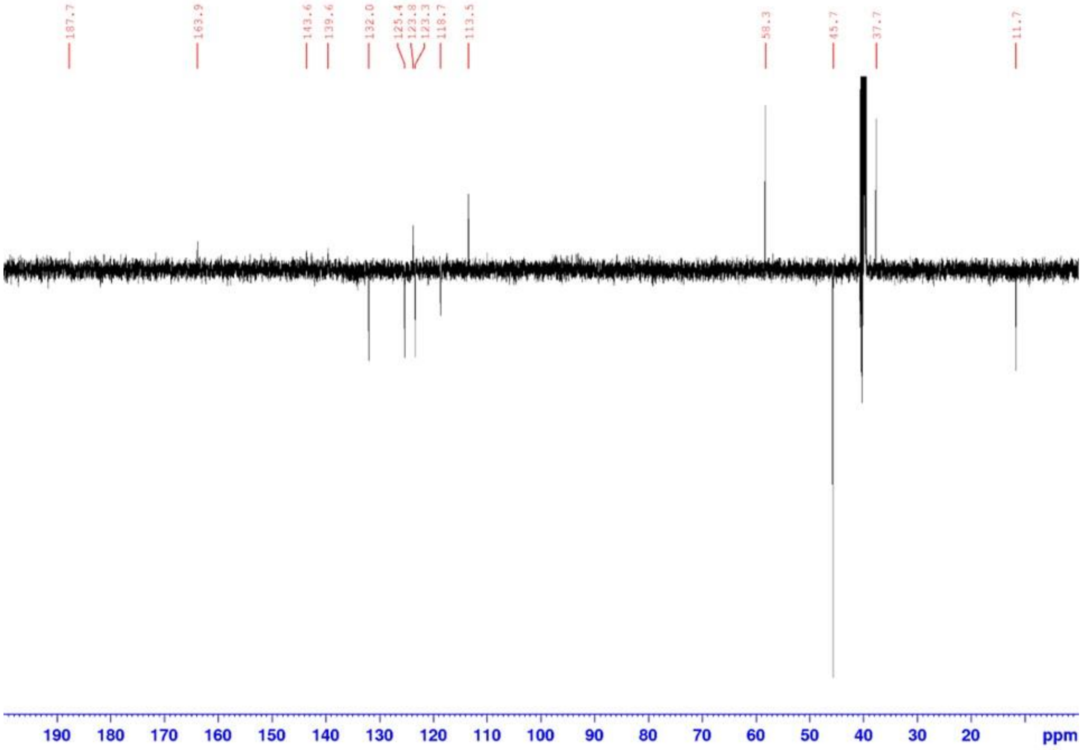


**Figure S4.** $^{13}$C-NMR spectrum of *N*-(2-(dimethylamino)ethyl)-3-methyl-4-oxo-1,4-dihydroquinoline-2-carboxamide

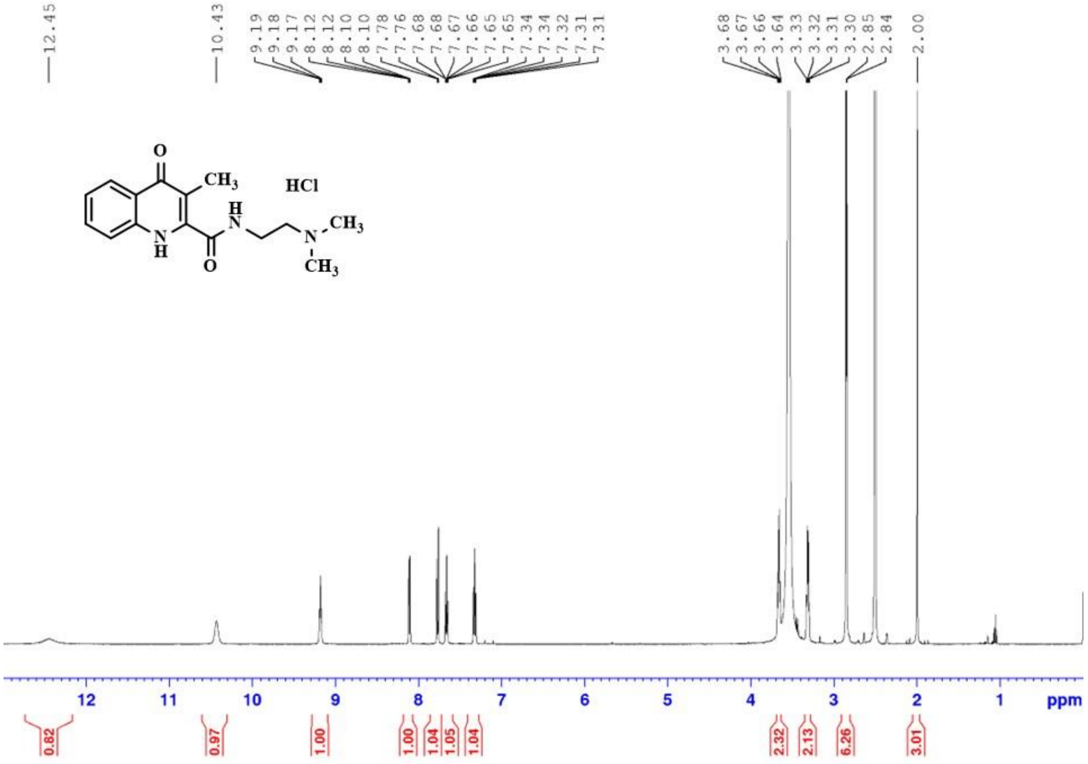

**Figure S5.** $^{1}$H-NMR spectrum of SZRG-21

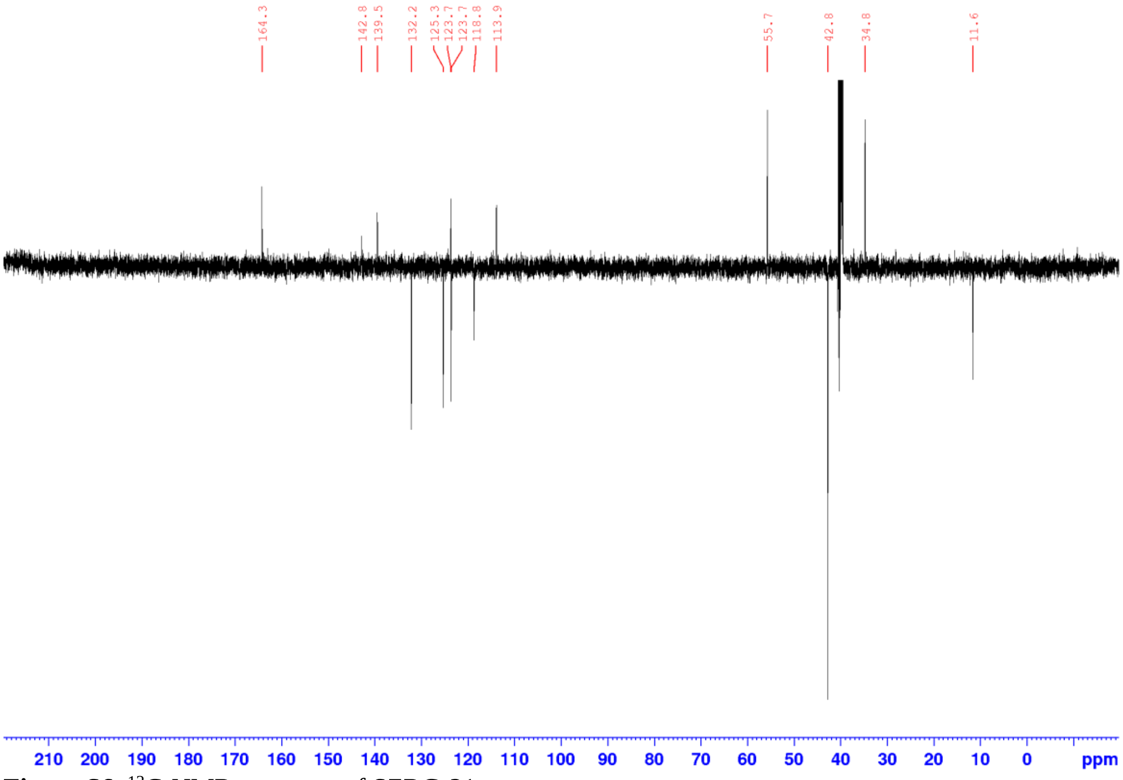

**Figure S6.** $^{13}$C-NMR spectrum of SZRG-21
